# Biogeographical Boundaries, Functional Group Structure and Diversity of Rocky Shore Communities along the Argentinean Coast

**DOI:** 10.1371/journal.pone.0049725

**Published:** 2012-11-16

**Authors:** Evie A. Wieters, Christopher McQuaid, Gabriela Palomo, Paula Pappalardo, Sergio A. Navarrete

**Affiliations:** 1 Department of Zoology and Entomology, Rhodes University, Grahamstown, South Africa; 2 Estación Costera de Investigaciones Marinas and Center for Marine Conservation, Pontificia Universidad Católica de Chile, Santiago, Chile; 3 Museo Argentino de Ciencias Naturales, Conicet, Buenos Aires, Argentina; The Australian National University, Australia

## Abstract

We investigate the extent to which functional structure and spatial variability of intertidal communities coincide with major biogeographical boundaries, areas where extensive compositional changes in the biota are observed over a limited geographic extension. We then investigate whether spatial variation in the biomass of functional groups, over geographic (10′s km) and local (10′s m) scales, could be associated to species diversity within and among these groups. Functional community structure expressed as abundance (density, cover and biomass) and composition of major functional groups was quantified through field surveys at 20 rocky intertidal shores spanning six degrees of latitude along the southwest Atlantic coast of Argentina and extending across the boundaries between the Argentinean and Magellanic Provinces. Patterns of abundance of individual functional groups were not uniformly matched with biogeographical regions. Only ephemeral algae showed an abrupt geographical discontinuity coincident with changes in biogeographic boundaries, and this was limited to the mid intertidal zone. We identified 3–4 main ‘groups’ of sites in terms of the total and relative abundance of the major functional groups, but these did not coincide with biogeographical boundaries, nor did they follow latitudinal arrangement. Thus, processes that determine the functional structure of these intertidal communities are insensitive to biogeographical boundaries. Over both geographical and local spatial scales, and for most functional groups and tidal levels, increases in species richness within the functional group was significantly associated to increased total biomass and reduced spatial variability of the group. These results suggest that species belonging to the same functional group are sufficiently uncorrelated over space (i.e. metres and site-to-site ) to stabilize patterns of biomass variability and, in this manner, provide a buffer, or “insurance”, against spatial variability in environmental conditions.

## Introduction

Boundaries between geographically distinct biotas are shaped and promoted by historical and ecological processes that set limits to the distribution of a large fraction of species. Transition zones are therefore areas of intense abiotic and biotic stresses modulated by limited dispersal, which limits colonization of habitat beyond a species range, and by vicariance processes that divide previously homogeneous genetic entities [1]. But beyond this broad understanding, we still have scarce information about whether geographic areas of extensive changes in species composition are also the geographic points where major changes at the population and community levels can be expected. That is, whether the same suite of processes generating range endpoints also affect, on the one hand, the populations of species that do cross these boundaries and, on the other hand, the functional structure of these communities. If these biogeographic regions permeate at all levels of organization, then management and conservation policies would be greatly facilitated as they could focus on these regions as priority areas to preserve, from genetic diversity to ecosystem function [2], [3], [4].

At the population level, a few studies have compiled information on the genetic structure of multiple species to examine whether genetic differentiation varies significantly with the presence of biogeographical barriers in the ocean. The answers have been mixed. For instance, on the Atlantic coast of North America, phylogeographic subdivisions of marine and freshwater species coincide with major bioegeographical breaks [5]. On the Pacific coast of North America, the well established biogeographical break at Point Conception does not correspond with phylogeographical breaks of most but a few species [6], [7], whereas major genetic differentiation has been detected at Cape Mendocino, a place of little compositional changes [4].

The question of whether the functional structure of communities (e.g. absolute and relative abundances of macroalgae, herbivores, etc.) changes abruptly across biogeographical breaks has been infrequently addressed, and rarely with a systematic sampling protocol. Indeed, spatially extensive field surveys of abundance of multiple species at all trophic levels are necessary to examine variation in functional structure across biogeographical provinces. A few published studies allow such evaluation (Table 1). For example, along the west and east coasts of southern Africa, Bustamante and Branch [2] found that major changes in functional group abundances were tightly associated to large-scale biogeographic transitions. But at a finer scale, on the west coast of South Africa, Wieters et al. [3] found important functional differentiation within a homogenous biogeographical region. Along the Pacific coast of central Chile, Broitman et al. [8], and see also [9], identified major changes in functional group structure of rocky shore communities geographically associated to an area of compositional changes in the biota [10], which in this case also coincides with the genetic differentiation of some species [11], [12], [13]. In contrast, similar large changes in abundances of functional groups documented at the limit between Oregon and California along the Pacific coast of North America [14] were not associated with biogeographic transitions and appear to be explained by abrupt regional differences in recruitment rates of several key species [15]. Through extensive field surveys along the entire coast of New Zealand, Schiel [16] showed that west and east coast biogeographic provinces are dominated by strikingly different functional groups (filter feeders and macroalgae, respectively), but no abrupt changes in the their abundances were associated to the Cook Straight biogeographic boundary separating the north and south islands. A comparison between the eastern and western coasts of the North Atlantic Ocean suggests that general patterns of community structure are similar between these shores, although the species richness within the functional groups and general processes might differ [17]. However, sampling along the shores was not sufficient to evaluate coincidence with biogeographic breaks.

**Table 1:** Summary of documented changes in abundance of functional groups and their coincidence (yes/no) with biogeographic limits.

Country	Location	Biogeographic Limit	Functional Group					Reference
			Macroalgae	Filter Feeders	Herbivores	Predators	Other	
South Africa	West-South Coasts	Boundary	Yes	Yes	Yes	Yes		Bustamante & Branch 1996
South Africa	South-East Coasts	Boundary	Yes	No	No	No		Bustamante & Branch 1996
South Africa	West Coast	None					*Yes	Wieters et. al. 2009
Chile	central coast	Transition	Yes	Yes	No	No		Broitman et al. 2001
United States	Oregon-California	None	Yes	Yes	NA	NA		Connolly & Roughgarden 1998
New Zealand	East-West coasts	Boundary	Yes	Yes	?	?		Schiel 2011
New Zealand	North-South Island: west coast	Boundary	No	No	?	?		Schiel 2011
New Zealand	North-South Island: east coast	Boundary	No	No	?	?		Schiel 2011
Argentina	Argentina-Magellanic	Boundary	No	No	No	No		This study

* Multivariate characterization of community structure based on biomass of functional groups.

NA = not evaluated.

? = unknown.

Interest in the functional structure of natural communities has increased significantly over the past decade, mainly because changes in species composition can deeply alter ecosystem functions and their resilience to environmental fluctuations [18], [19], [20]. Loss of resilience can then lead to further and potentially devastating collapses of biodiversity and further loss of ecosystem function [20], [21], [22], [23]. Thus, there is growing interest in the “functional performance” of natural assemblages and whole communities - how efficient (i.e. in depleting a resource, in contributing to primary productivity or standing stock), resilient and/or persistent their functional structures are in the face of environmental fluctuations. A number of hypotheses relating diversity and “functional performance” have been advanced [19], [24], [25], and experimental manipulations conducted in the laboratory, in mesocosms or under field conditions have been the main approach to evaluate them. Recent reviews of these findings strongly support the existence of a positive relationship between species richness and functional group standing stock, primary productivity or resource depletion [20], [26]. There is, however, uncertainty as to the mechanisms through which diversity affects ecosystem function and how these results scale up to landscape and regional levels and generalize across ecosystem types and processes [24], [26]. Indeed, one of the mechanisms through which species richness can increase ecosystem function is through providing an insurance (i.e. increased resilience or resistance) in the face of temporal or spatial variation in environmental conditions; the “insurance hypothesis” [27]. However, the levels of environmental variability are limited to conditions recreated in the experiments. Another mechanism through which diversity can increase ecosystem function is by simply increasing the probability that a critical species (e.g. “keystone species”) forms part of the assemblage; the “sampling effect” hypothesis [28]. But these probabilities are preset by the pool of species chosen for the experiment. Therefore, examination of the relationships between diversity and biomass or productivity of functional groups across biogeographical regions, i.e. across natural levels of environmental variability and species composition, can shed light into the commonality of this relationship in nature.

**Figure 1:**
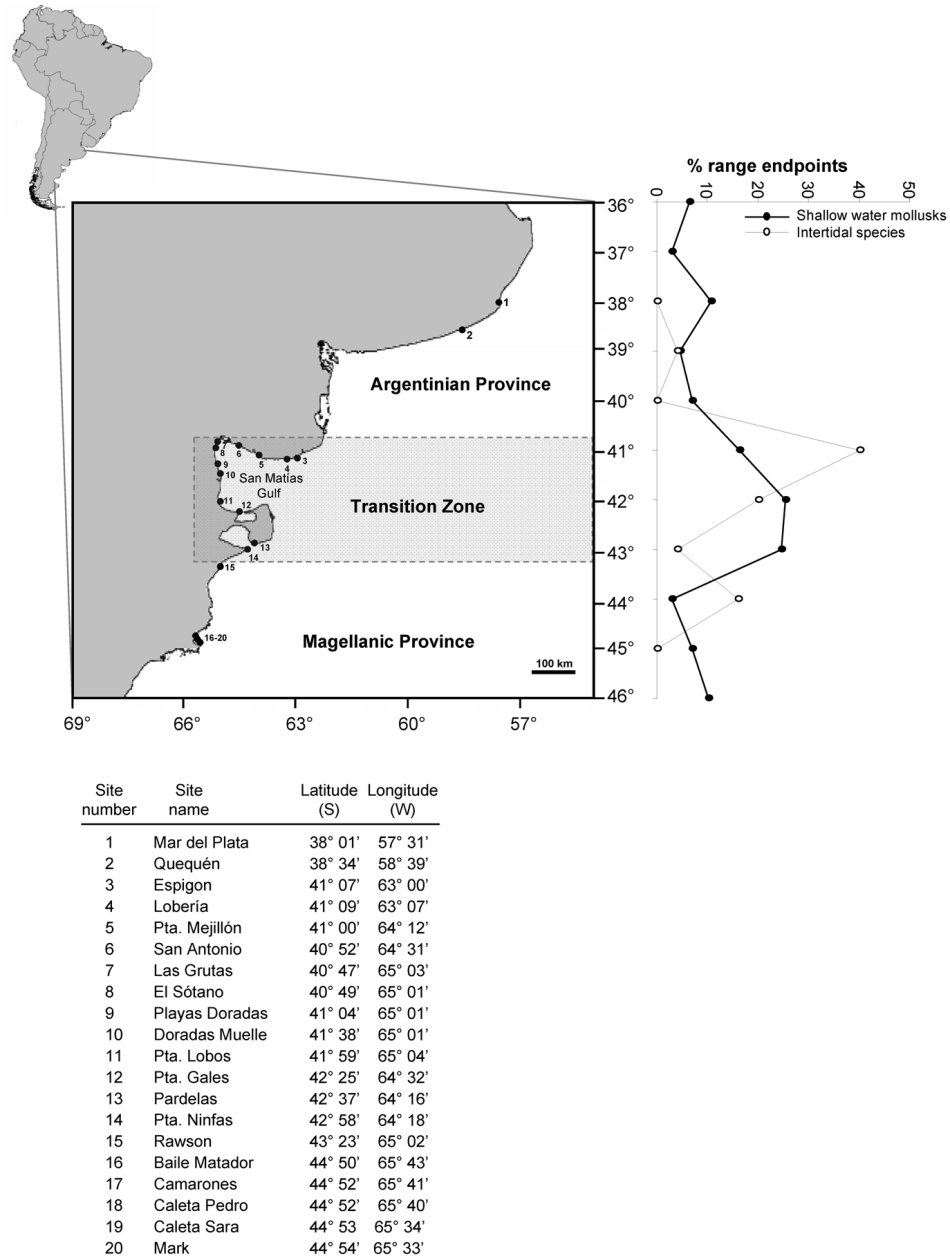
Map of Argentina and study region indicating the positions of survey sites and the distribution of species range limits. Numeric codes for sites as used in other figures with corresponding location details listed. Province and transition zone borders are indicated by dashed lines. Shaded region indicates transition zone. The distribution of species range limits along the study region: Data for shallow water mollusks are from a database of biogeographical distributions of more than 932 species along the southwest Atlantic from 10 to 55°S (P. Pappalardo, unpublished data). Distribution data compiled from intensive literature search. The figure plots the percentage of species with southern and northern range endpoints for each latitudinal band (1 degree bins) between 36 and 46°S, the limits of our study region. Data for intertidal invertebrates and algae come from the one-time field surveys described in this study. The peak of range boundaries occurs in the transition zone.

**Table 2:** List of identified genera assigned to the different functional groups.

Articulated	Corticated	Filter Feeders	Grazers
*Bossiella*	*Adenocystis*	*Amiantis*	*Arbacia*
*Corallina*	*Ahnfeldtia*	*Amphibalanus*	*Fissurella*
*Jania*	*Ahnfeltiopsis*	*Aulacomaya*	*Nacella*
	*Antithamnion*	*Balanus*	*Plaxiphora*
**Ephemeral**	*Aphanocladia*	*Brachidontes*	*Siphonaria*
*Blidingia*	*Asperococcus*	*Bryozoa*	*Tegula*
*Bryopsis*	*Ballia*	*Crepidula*	*Tonicia*
*Callithamnion*	*Bostrichia*	*Halichondria*	
*Ceramium*	*Chondria*	*Hydrozoa*	**Predators**
*Chaetomorpha*	*Codium*	*Hymeniacidon*	*Anasterias*
*Cladophora*	*Colpomenia*	*Molgula*	*Cyrtograpsus*
*Ectocarpus*	*Cordariopsis*	*Mytilus*	*Halicarcinus*
*Enteromorpha*	*Dictyota*	*Ostrea*	*Nudibrancha*
*Hincksia*	*Gigartina*	*Perumytilus*	*Pareuthria*
*Polysiphonia*	*Gymnogongrus*	*Phragmatopoma*	*Trophon*
*Porphyra*	*Mazzaella*	*Retrotapes*	
*Scytosiphon*	*Nothogenia*	*Tedania*	**Scavengers**
*Streblocladia*	*Petalonia*		*Buccinanops*
*Ulva*	*Plocamium*		*Pagurus*
	*Rhodymenia*		*Loxopagurus*
	*Schizymenia*		*Turbonilla*
	*Undaria*		

Here, we follow a two-prong approach that lays a bridge between biogeography and ecology. First, we describe geographical variation in the functional structure of rocky intertidal communities along much of the coast of Argentina and evaluate the hypothesis that major changes in the absolute and relative abundance of functional groups match traditional, well-known biogeographic boundaries (i.e. regions of rapid compositional changes). Second, across this region we evaluate whether: a) increased species richness leads to increased functional group biomass, and b) increased species richness leads to reduced susceptibility to environmental fluctuations, as estimated by the level of variability in functional group biomass over scales of tens of metres within sites, and over geographical scales of hundreds of kilometres among sites.

**Figure 2:**
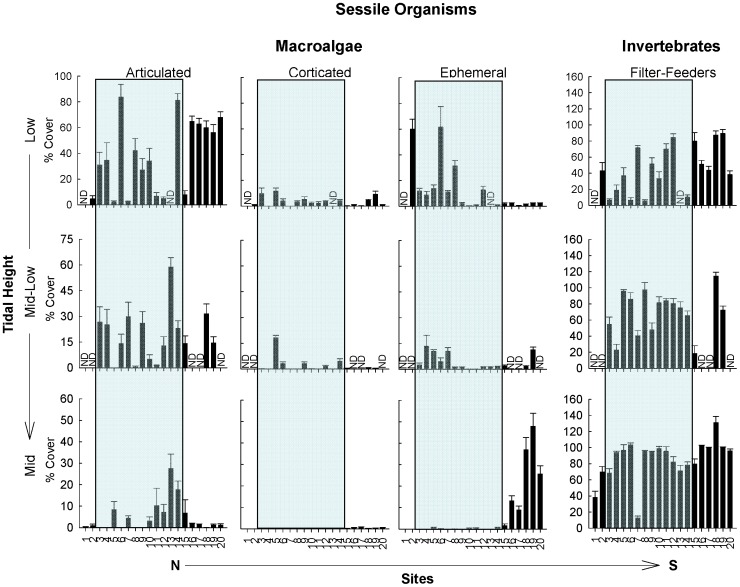
Mean percentage cover (± SE) of dominant sessile functional groups of the low, mid-low, and mid intertidal zones. Note the different y-axis scales used. The shaded background indicates those sites within the biogeographical transition zone. ND = no data.

**Figure 3:**
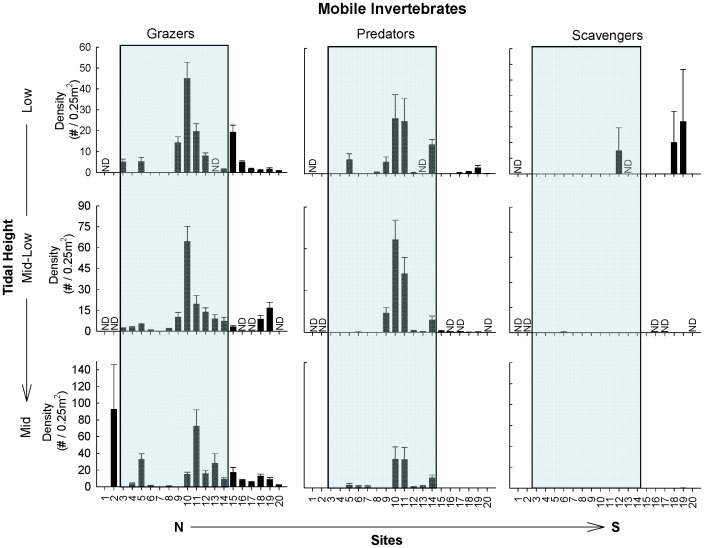
Mean density per 0.25 m^2^ (± SE) of dominant mobile functional groups of the low, mid-low, and mid intertidal zones. Note the different y-axis scales used. The shaded background indicates those sites within the biogeographical transition zone. ND = no data.

**Table 3:** Nested ANOVA tests of functional group abundances in relation to Region (Argentinean Province, Magellanic Province and Transition Zone) and among-site differences for a) low, b) mid-low, and c) mid intertidal zones.

	df	MS	F	P
a) *Low Zone*				
**Articulated**				
Region	2	22902.9	3.13	0.071
Site (Region)	15	10961.7	12.10	**<0.0001**
Error	382	905.7		
**Corticated**				
Region	2	131.42	1.14	0.342
Site (Region)	15	168.23	7.84	**<0.0001**
Error	382	21.45		
**Ephemeral**				
Region	2	18092.7	12.54	**0.0006**
Site (Region)	15	2218.41	28.44	**<0.0001**
Error	382	78.01		
**Filter Feeders**				
Region	2	26962.4	3.10	0.073
Site (Region)	15	13128.2	14.15	**<0.0001**
Error	382	928.0		
**Grazers**				
Region	2	817.43	0.66	0.53
Site (Region)	15	1928.19	36.40	**<0.0001**
Error	379	52.98		
**Predators**				
Region	2	40.45	2.58	0.10
Site (Region)	15	22.83	7.09	**<0.0001**
Error	379	3.22		
**Scavengers**				
Region	2	0.0014	0.14	0.87
Site (Region)	15	0.0109	1.36	0.17
Error	379	0.0081		
b) *Mid-Low Zone*				
**Articulated**				
Region	1	41.33	0.014	0.91
Site (Region)	13	3254.89	6.34	**<0.0001**
Error	171	513.21		
**Corticated**				
Region	1	118.25	0.84	0.38
Site (Region)	13	156.02	27.05	**<0.0001**
Error	171	5.77		
**Ephemeral**				
Region	1	17.48	0.07	0.79
Site (Region)	13	254.46	4.73	**<0.0001**
Error	171	53.81		
**Filter Feeders**				
Region	1	242.47	0.03	0.87
Site (Region)	13	9494.54	12.00	**<0.0001**
Error	171	791.00		
**Grazers**				
Region	1	93.02	0.04	0.84
Site (Region)	13	2329.4	16.97	**<0.0001**
Error	176	137.28		
**Predators**				
Region	1	339.30	1.06	0.32
Site (Region)	13	352.35	24.70	**<0.0001**
Error	176	14.26		
c) *Mid Zone*				
**Articulated**				
Region	2	1072.66	1.66	0.22
Site (Region)	17	820.34	6.10	**<0.0001**
Error	423	134.40		
**Ephemeral**				
Region	2	11970.5	3.95	**0.04**
Site (Region)	17	3948.64	12.17	**<0.0001**
Error	423	324.49		
**Filter Feeders**				
Region	2	6.84	2.82	0.086
Site (Region)	17	3.18	16.59	**<0.0001**
Error	423	0.19		
**Grazers**				
Region	2	12841.9	2.27	0.13
Site (Region)	17	7287.9	6.72	**<0.0001**
Error	430	1085.24		
**Predators**				
Region	2	15.35	3.08	0.07
Site (Region)	17	6.42	6.87	**<0.0001**
Error	430	0.93		

Bold values are significant at α = 0.05.

### The System: Southwest Atlantic

The study region spans 6 degrees of latitude, from Mar del Plata (38° 00′ S, 57° 31′ W) to Cabo Dos Bahías (44° 55′ S, 65° 31′ W) and extends across the marked boundary at 43°S that divides two major coastal biogeographical provinces, the Argentinean and Magellanic provinces (Fig. 1). For nearshore organisms, the area from 41–43°S that includes the north-Patagonic Gulfs and Peninsula Valdés is generally considered to be a strong transition zone between these two provinces (Fig. 1). Like most places in the world, determination of biogeographical regions and provinces has been largely based on species range boundaries for individual taxa together with characterization of oceanographic/environmental conditions. In the case of Argentina, these limits appear to follow discontinuities in oceanographic characteristics, namely the converge of the Brazil-Malvinas currents, which produce extreme temperature gradients [29], [30], [31]. However, other authors have associated the biogeographic provinces with changes in productivity and habitat features [32].

**Figure 4:**
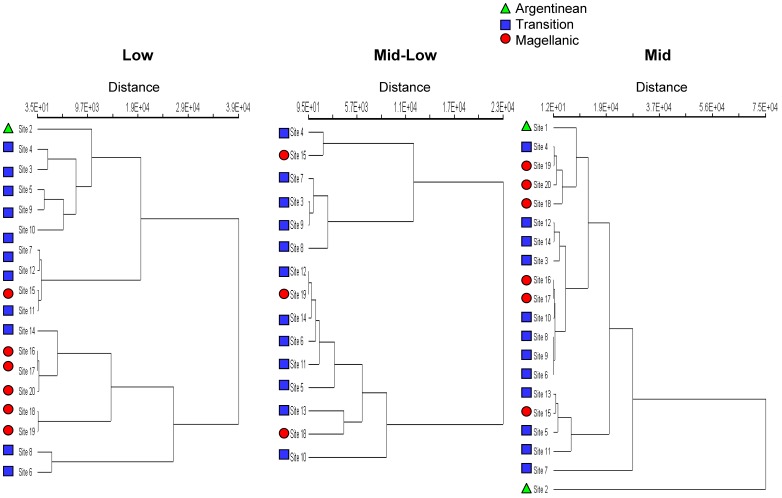
Dendogram depicting sites numbered 1–20 from north to south for low, mid-low, and mid intertidal zones. Biogeographical regions are coded by coloured symbols.

The Argentinean Province includes coastal and inner shelf waters from Rio Grande do Sul at 30–32°S to the southern limit in northern Patagonia at Peninsula Valdés – Rawson, Chubut; Argentina [29], [30]. The region is characterized by the poleward flow of the Brazil Coastal Current that carries warm tropical waters, mixed with continental runoff originating largely from Rio de la Plata and the Lago dos Patos. Moveable and suspended sandy sediments, interspersed with isolated hard-bottom habitats, predominate throughout the province to eventually give way to more consistent consolidated surfaces in the transition zone and further south. Typically, winds predominate from the north in this region [29], [30].

**Figure 5:**
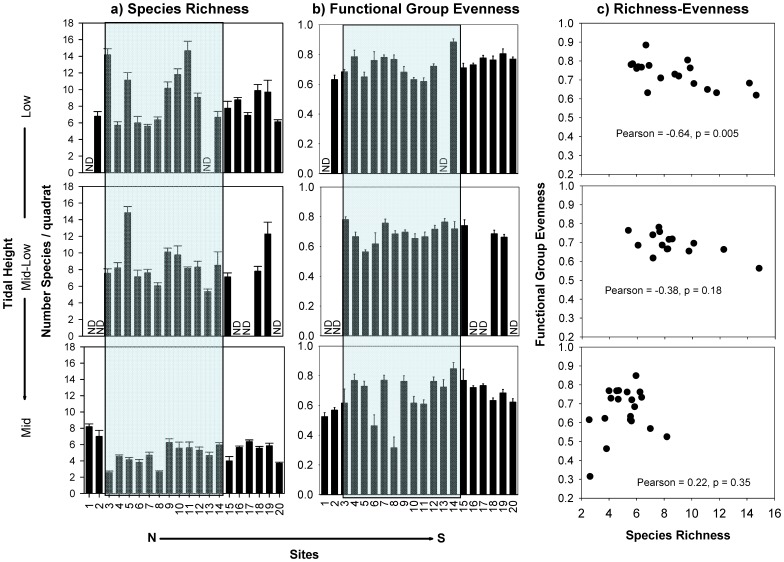
Mean a) number of species and b) Peilou’s functional group evenness index per 0.25 m^2^ quadrat (± SE) across the low, mid-low, and mid intertidal zones. ND = no data. Scatterplots of mean species richness and evenness for functional groups. The shaded background indicates those sites within the biogeographical transition zone.

**Figure 6:**
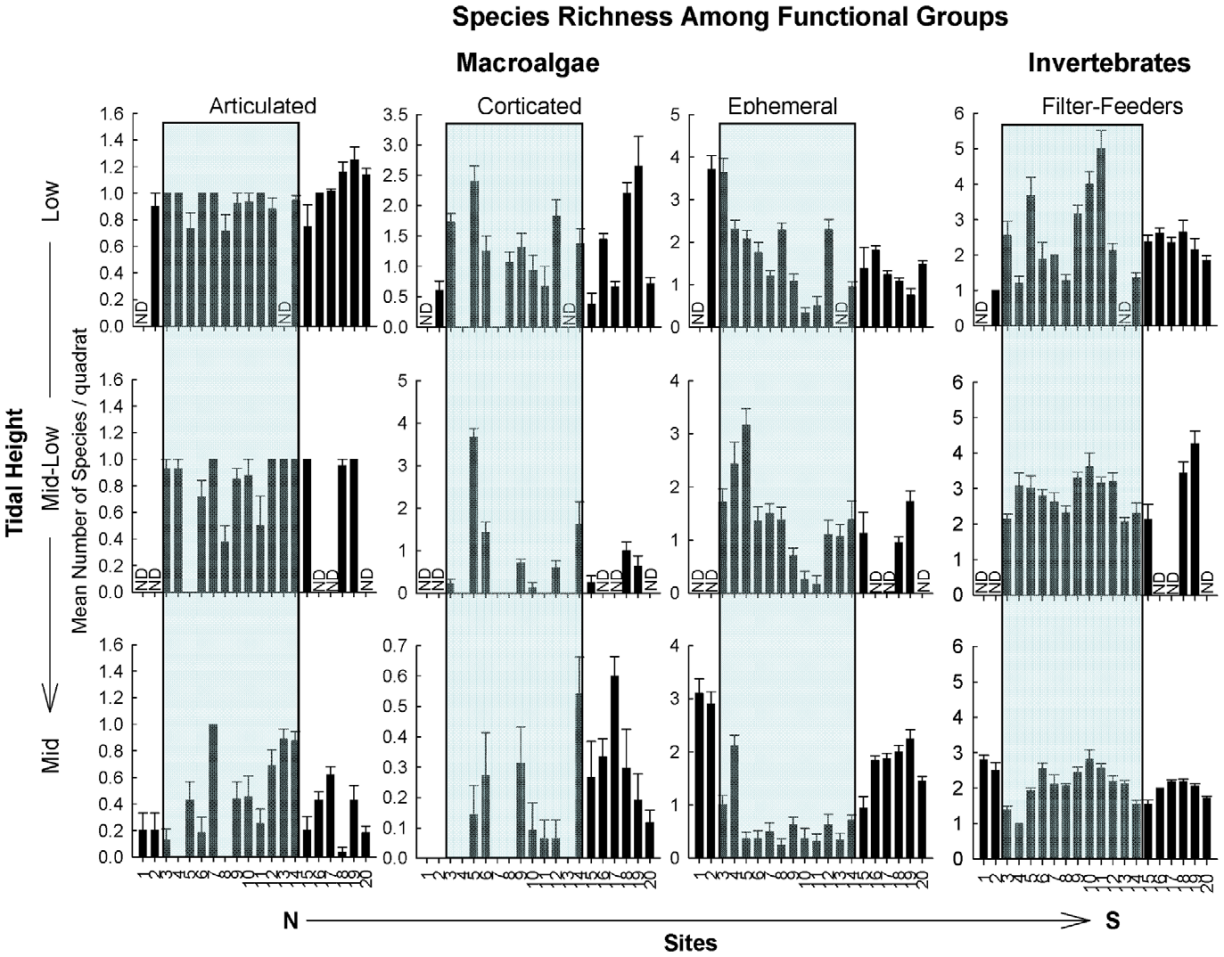
Mean number of species per 0.25 m^2^ (± SE) of dominant sessile functional groups of the low, mid-low, and mid intertidal zones. Note the different y-axis scales used. The shaded background indicates those sites within the biogeographical transition zone. ND = no data.

**Figure 7:**
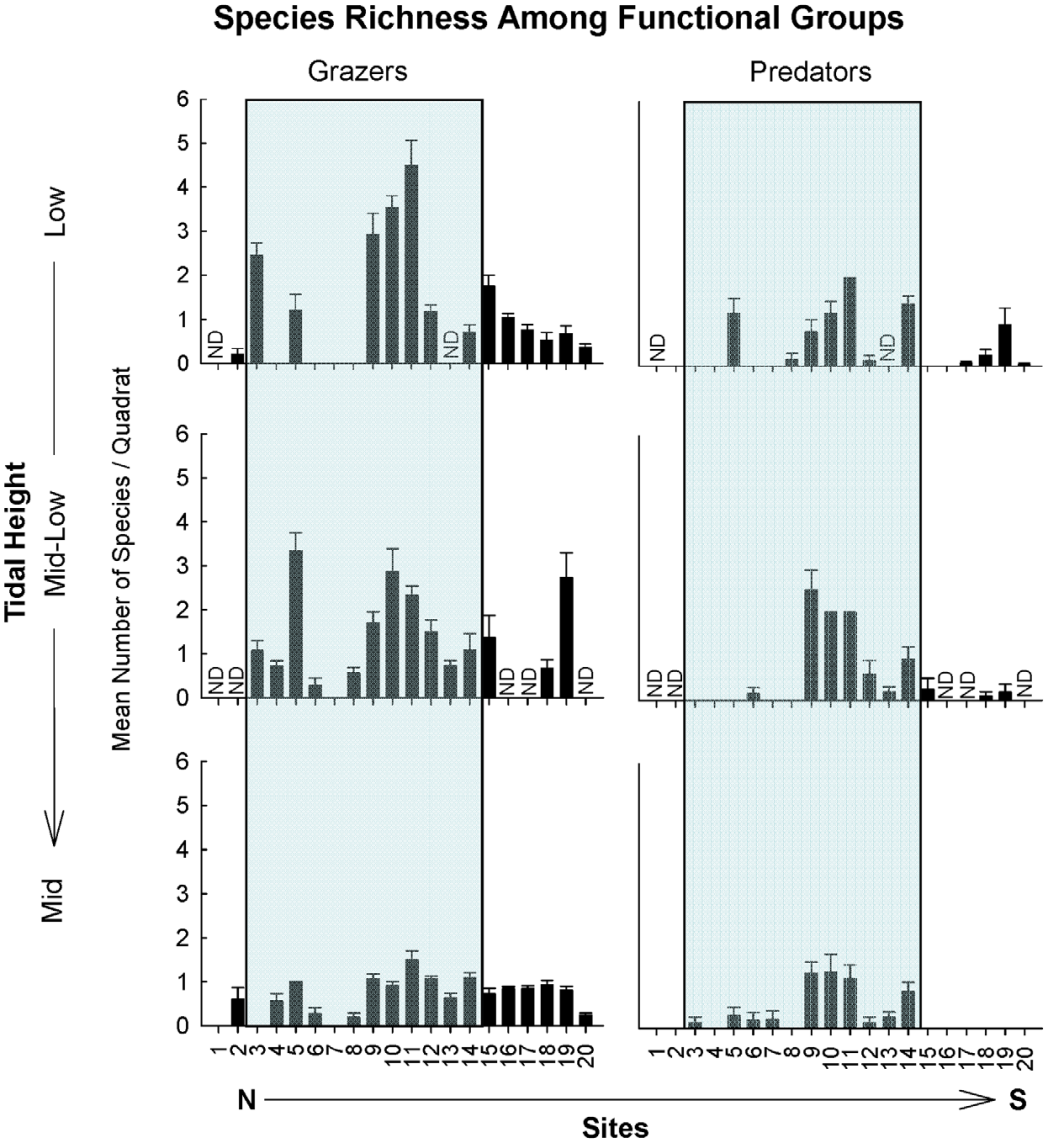
Mean number of species per 0.25 m^2^ (± SE) of dominant mobile functional groups of the low, mid-low, and mid intertidal zones. Note the different y-axis scales used. The shaded background indicates those sites within the biogeographical transition zone. ND = no data.

**Table 4:** Nested ANOVA tests of overall species richness in relation to region (Argentinean Province, Magellanic Province and Transition Zone) and among-site differences for a) low, b) mid-low, and c) mid intertidal zones.

	df	MS	F	p
**Low Zone**				
Region	2	50.69	0.67	0.52
Site (Region)	15	113.49	12.74	**<0.0001**
Error	379	8.91		
**Mid-Low Zone**				
Region	1	9.59	0.17	0.68
Site (Region)	13	58.88	7.73	**<0.0001**
Error	171	7.62		
**Mid Zone**				
Region	2	80.01	3.36	0.06
Site (Region)	17	31.12	13.84	**<0.0001**
Error	423	2.25		

Bold values are significant at α = 0.05.

The Magellanic Province has its northern coastal limit near Rawson (Fig. 1). To the south it extends to the southern tip of the continent and wraps around the continent along the Pacific coast north of Chiloé Island, Chile [33]. On the Atlantic side, the region is characterized by dominance of cold subantarctic waters and species. South of 40°S, the Patagonian shelf (defined by the 200 m isobath) widens progressively, from 170 km at 39°S to 850****km at about 50°S. This region is characterized by anomalously large tidal currents, with tidal ranges nearing 12 m. Marked tidal fronts have been described near the Valdés Península and San Jorge Gulf [34]. A region of low sea surface temperature surrounds Peninsula Valdés and extends southward along the coast [35], [36], with high chlorophyll concentrations in the inner shelf and near the coast in the spring-summer months [37]. Peninsula Valdés also marks a spatial shift in seasonal variability of surface chlorophyll, with bloom initiation in early austral spring (Sept-Oct) to the north of 40–45°S, while to the south, blooms begin in late spring to early summer (Nov-Jan) [37].

**Table 5:** Correlations (Pearson) between a) mean species richness and local biomass and b) mean species richness and within-site spatial variation (coefficient of variation, CV, among quadrats) of biomass across the 20 sites surveyed for the low, mid-low, and mid intertidal zones.

	Low	Mid-Low	Mid
a) *Species Richness vs. Biomass*			
Articulated	**0.49** *	**0.55** *	**0.59** **
Corticated	**0.74** ***	**0.96** ***	**NA**
Ephemeral	**0.63** **	**0.24**	**0.51** **
Filter feeder	0.33	0.50	−0.15
Grazer	**0.76** ***	**0.76** ***	0.44
Predator	**0.84** ***	**0.77** ***	**0.92** ***
Scavenger	**0.51** *	**NA**	**NA**
b) *Species Richness vs. CV Biomass*			
Articulated	−**0.58** *	−**0.71** **	−**0.81** ***
Corticated	−0.35	−**0.83** **	**NA**
Ephemeral	−**0.62** **	−**0.50**	−**0.69** ***
Filter feeder	−0.32	−0.49	0.02
Grazer	−**0.78** ***	−**0.73** **	−**0.81** ***
Predator	−**0.80** **	−**0.84** **	−**0.93** ***
Scavenger	**−0.65** **	**NA**	**NA**

Bold values indicate significance at α = 0.05.

* p<0.05

** p<0.01

*** p<0.001.

NA indicates where the functional group was too scarce in a given tidal level for meaningful correlations.

At a smaller scale within the transition zone and San Matías Gulf (Fig. 1), a strong thermal front divides two markedly different zones, with relatively warmer and saltier waters of limited nitrate concentrations in the northwest, and lower temperature, low salinity and higher nitrate waters in the south and southeast [38], [39]. These differences are most pronounced in spring-summer [39].

**Figure 8:**
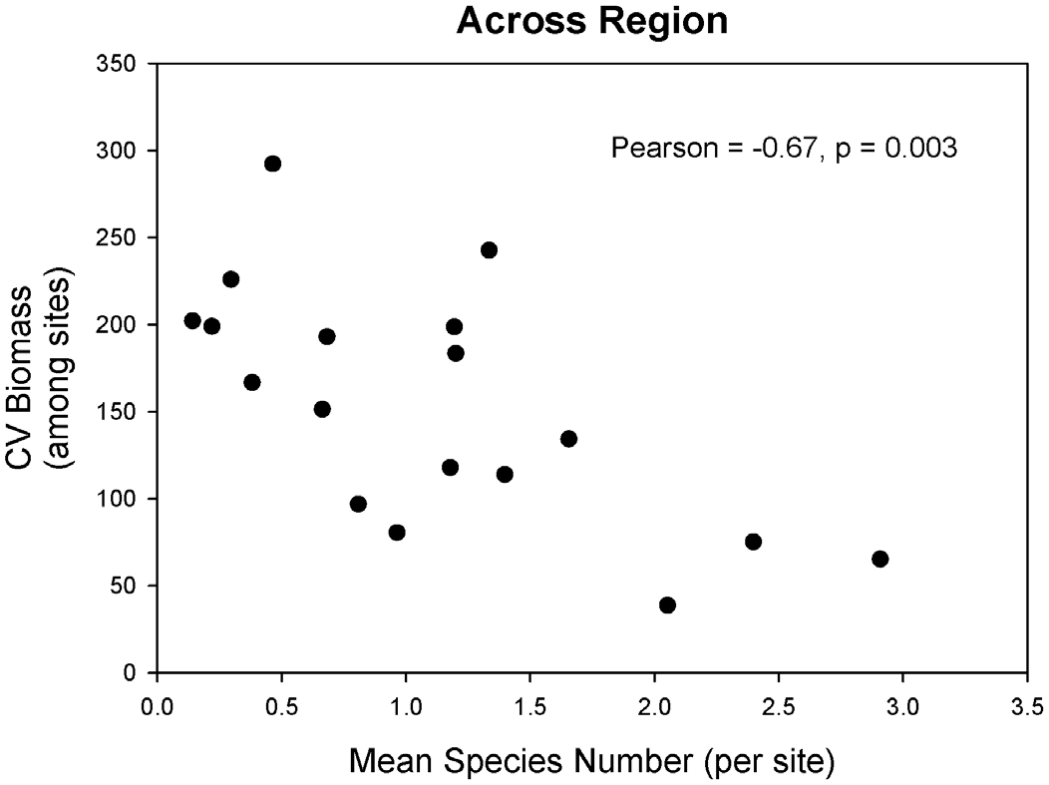
Relationship between mean species number across sites and the coefficient of variation of biomass among sites across the region for individual functional groups across the different intertidal zones.

## Methods

Intertidal communities at 20 sites, spaced 10****s–100****s km apart across the region were surveyed during austral winter-spring months of 2008 and 2009. Most sites were surveyed only once, but each year surveys included sites from all biogeographic provinces to try to avoid confounding between-years variability with regional differences. A few sites have been sampled on multiple years and they showed little variation at the level of functional group abundance. Due to the limited rocky shore habitat north of 41°S, it was only possible to sample 2 sites (site 1 - Mar del Plata and site 2 - Quequén) in the northern section of the study region. Sampling was more intensive in the biogeographic transition zone. Sites were composed of gently sloping (0–20°) rocky benches openly exposed to oceanic swells and were selected based on availability of similar suitable rocky habitat and accessibility. The two most southern sites (Caleta Sara and Marc) fall within the Cabo Dos Bahias nature reserve, which primarily aims to protect local colonies/rookeries of marine mammals (e.g. sea lions and elephant seals) and, secondarily, birds (e.g. penguins). Surveys were based on the transect-quadrat sampling method, similar to that described in Wieters et al. [3] and Broitman et al. [8]. Percentage cover and density of macroscopic sessile and mobile species, respectively, were quantified in a minimum of eight and a maximum of 63 0.25 m^2^ quadrats haphazardly placed along 20 m –200 m transects stretched parallel to the shoreline at low, mid-low and mid intertidal zones of 1–3 rocky shores separated by 60–100 m at each study site. A single shore was sampled at 6 sites. The number of shores and quadrats surveyed varied in order to sample a similar proportion of area at each site. A species accumulation curve (number of species as a function of number of quadrats) constructed from 55 quadrats sampled in the mid intertidal zone at Mar del Plata showed that 6–7 quadrats provided good estimates of the local number of species in this generally species-poor system (see Supplemental Information, Fig. S1). However, a fraction of rare species could be under-sampled in our study. Due to local geomorphology and topography, not all zones were present at all sites. Cover of species inhabiting primary space (attached to rock surface) and secondary space (i.e. epibiotic such as barnacles on mussels) were estimated separately, so total cover could exceed 100%. Species present but covering less than 1% of quadrat area were scored as 0.5% cover. All organisms were resolved to the lowest possible taxonomic level in the field, collecting a few specimens for identification when necessary. To minimize potential taxonomic biases, 80% of quadrats were recorded by a single observer. Biomass (wet weight) of each species was calculated for each quadrat using site-specific conversions of cover to mass for sessile species and length:mass regressions for mobile species [3]. Transect length, distance between quadrats, and the number of shores sampled at each site depended upon local topography and area of available rocky habitat. Areas obviously influenced by sediment abrasion were avoided, as were tidepools. To concentrate on regional trends, data from transects/shores within a site and from 2008 and 2009 spring surveys were pooled after examining that general trends were similar between years.

We categorized species into one of 7 functional groups (Table 2) based on a) morphological traits related to ability to respond to nutrient availability (e.g. productivity potential) and resistance to herbivory (algae) or b) trophic level and foraging strategy (invertebrates) [3], [8]. For each tidal height (zone), abundances (percentage cover or density) and species richness of individual functional groups were separately analyzed with ANOVA. Region (Argentinean Province, Magellanic Province, and transition zone) was considered a fixed factor, whereas Site was considered a random factor nested within region. Quadrats were then considered a random factor nested within Site. When necessary, data were transformed to meet model assumptions, which were checked by visual inspection of residuals. We used Mantel tests to examine the correlation between the matrices of functional group structure and coastal geographical distances among sites, separate for each intertidal zone.

Multivariate characterization of variability in functional structure across the study region was performed with Bray-Curtis ordination with abundance of the different functional groups as the dependent response variables. Prior to analysis, data were standardized to the most abundant group to place emphasis on relative changes across the region. Bray-Curtis ordination provided the best representation of data compared to other non-parametric techniques, such as NMDS. To further define grouping of sites based on functional group similarity, we conducted a hierarchical cluster analysis based on Euclidean distance and Ward’s method of group linkage in PC-Ord Software (McCune & Mefford, 2009, V 6.1, MjM Software, Gleneden Beach, Oregon, U.S.A.). Bootstrapping was used to define the cut off level for significant clusters.

Local species richness was estimated as the average number of species observed within a quadrat at each site, as well as the sum total number of species observed within a zone at each site, both across all functional groups and within each functional group. Since our focus was on richness of species comprising the main functional groups observed in the community, our estimates do not include cryptic species or micro-habitat specialists that can only be observed through destructive sampling. To examine to what extent local richness was equally distributed among the different functional groups (functional group evenness), we calculated Peilou’s evenness index per quadrat based on the number of species per functional group. We then examined regional patterns in species richness within the study region and their correspondence with biogeographical boundaries. The relationships between species richness within a functional group and either total biomass of the functional group, or spatial variability in biomass of the functional group, measured as coefficients of variation among quadrats (meters apart) or among sites, were examined through pairwise correlations. Caution and potential artifacts in these relationships are presented in the Discussion.

To further illustrate and define the geographic location of these bioegeographical provinces, and since there is no published record showing species range endpoints for the coastal marine biota in the study region, we compiled from the literature a database containing records of geographic distribution of 932 species of shallow water benthic invertebrates. This database forms part of another study by P. Pappalardo and details will be published elsewhere. We also examined range endpoints in our more restricted database of benthic invertebrates. Patterns of macroalgal species richness and composition turnover were recently presented by Liuzzi et al. [31], who document a large discontinuity coincident with these same biogeographic province limits, even after accounting for changes/biases in sampling effort.

## Results

Range endpoints of coastal benthic invertebrates from literature compilations generally corroborate qualitative description of biogeographic provinces (Fig. 1b). Similarly, a large fraction of intertidal species in our database find their geographic endpoints within the reported biogeographic boundaries (Fig. 1b), supporting again the occurrence of significant compositional changes within the region.

### Abundance of Individual Functional Groups

Variation in alongshore patterns in the abundance of each functional group across all tidal levels were generally not related to previously defined biogeographical regions or to the major physical-oceanographic features previously described within the transition zone (Figs. 2, 3; Table 3). Nevertheless, there were a few strong geographical trends at specific tidal elevations. Most notably, ephemeral algae presented abrupt, discontinuous and significant changes across the regions that differed markedly across tidal elevations (Fig. 2, Table 3). Along the mid shore, ephemeral algae (mostly *Porphyra* and *Ulva*) sharply increased in abundance from being a minor element in the functional structure of communities throughout the Argentinean Province and transition zone, to being one of the most abundant functional groups in the Magellanic Province. Interestingly, a contrasting pattern was observed in the low zone, where ephemeral algae (mostly filamentous turfs; e.g. *Polysiphonia*, *Ceramium*) were consistently more abundant north of El Sótano (site 8). However, this smaller discontinuity occurred in the middle of the transition zone, northwest corner of San Matías Gulf, and did not coincide with the limits of main biogeographic provinces (non-significant effect of Region, Table 3).

In addition to the abrupt regional changes described above, a few other geographical patterns of functional structure were observed. Articulated algae occupying the mid intertidal zone were more frequently abundant towards the southern end of the transition zone, (sites 11–14 between Pta. Lobos and Pta. Ninfas, Fig. 2). Herbivore grazers (e.g. the limpets *Siphonaria*, *Nacella* and chitons) and the generally scarce carnivorous predators (e.g. predominantly the gastropod *Trophon*) presented marked peaks in abundance at the center of the transition zone (site 9–11, between Playas Doradas and Pta Lobos, Fig. 3), but again, there were no strong changes in the abundances of these functional groups at the boundaries with either the Argentinean or the Magellanic provinces (non-significant effect of Region at all tidal heights, Table 3). These alongshore patterns were remarkably similar across tidal heights.

Due to lack of suitable habitat, our limited sites in the Argentinean Province were also further away from the others than we would have liked and thus may have increased potential for confounding effects. However, Mantel tests showed that geographical distance among sites did not correlate with distance in functional group structure for low (Mantel r = 0.106, p = 0.216), mid-low (Mantel r = −0.043, p = 0.648), or mid (Mantel r = 0.077, p = 0.441) intertidal zones.

### Community-level Spatial Structure and Similarity

The multivariate characterization of functional group abundances based on Bray-Curtis ordination showed marked differentiation (spatial structure) among groups of sites, but importantly, there was again no clear effect of geographical configuration of the coastline or latitudinal patterning (see Fig. S2 in Supporting Information). In other words, when examining variation in all functional groups together, sites did not show obvious similarity with geographical neighbours. Consequently, multivariate groupings of sites did not conform to biogeographical regions. The same was true for the results of cluster analyses; there was spatial structure at all shore levels, but this failed to correspond to biogeographical regions or to follow a latitudinal gradient (Fig. 4).

### Species Richness and Functional Group Biomass and Variability

No clear geographical (latitudinal) patterns emerged in average species richness in 50 × 50 cm quadrats, nor did we detect abrupt changes in richness at biogeographical transition zones or limits (Fig. 5–7). The total number of species present at a site (simply summed across quadrats) presented only a tendency to increasing richness to the south in the low and mid intertidal zones, but again no abrupt changes were associated with biogeographical limits (see Figure S3 in Supporting Information). Mean species richness per quadrat was similar across the different regions for all tidal heights (non-significant effect of Region, Table 4). Detailed analyses of species richness using a variety of parametric and non-parametric diversity estimators will be presented in a separate publication.

The distribution of species across functional groups (functional group evenness) showed lower among-site variation across the coast (CV among sites: low = 9.7, mid-low = 8.3, mid = 19.1) than did species richness, particularly in the low and mid-low intertidal zones (Fig. 5). In the low intertidal zone, the total number of species at a given site was strongly and negatively correlated with the degree to which a single functional group dominated community composition (Fig. 5c). In contrast, species richness was unrelated to functional group evenness in the mid-low and mid intertidal zones.

The average number of species within functional groups was strongly, positively correlated with functional group biomass in all cases, except filter feeders (in all zones), ephemerals in the mid-low zone and grazers in the mid zone (Table 5a). Thus, increased species richness within a functional group generally led to increased biomass of that group. Similar significant positive relationships were observed when using cover and density as estimates of abundance (see Table S1 in Supplementary Information). The lack of dependence of filter feeder performance among sites on species richness is notable because this group was often the most dominant space-occupier on the shore, but there is marked tendency to dominance by a single species at a given tidal level. This also shows that in intertidal communities these variables are not inevitably and positively associated to sampling effects [28].

Local (10’s–100’s metres within a site) variance in functional group biomass standardized by the mean (CV’s) was strongly, negatively correlated with the average number of species within a functional group (Table 5b). The relationship was consistent across all functional groups and intertidal zones, with the exception of filter feeders, low shore corticated macroalgae, and ephemerals in the mid-low shore. Again, these results were robust to the manner in which abundance was estimated (see Table S1 in Supplementary Information). Similarly, a significantly negative relationship was also observed between regional (among-site) standardized variance in functional group biomass and species richness (Fig. 8). Thus, as we discuss below, increased species richness appears to stabilize variance in functional group biomass at both local and regional scales.

## Discussion

The extent to which the structure of communities, at the level of the major functional groups, is dependent on the processes that produce extensive compositional changes in the biota, such as those associated with biogeographical boundaries, has seldom been directly investigated (see Table 1). Here, we describe geographical variation in individual and multivariate characterizations of functional community structure for intertidal assemblages spanning six degrees of latitude and extending across the biogeographical limits between the Argentinean and Magellanic Provinces of the southwest Atlantic. We found marked spatial structure in rocky intertidal communities in terms of major functional groups, but patterns of abundance generally did not coincide with biogeographical limits. Thus, processes that determine the structure in functional groups of these intertidal communities appear to be distinct from those setting species range boundaries. For most functional groups, there was a significant positive relationship between the biomass of a group and species richness and a negative relationships between species richness and spatial variance in biomass at two different spatial scales. Thus, increasing the number of species within most functional groups appears to increase the ability to use resources more fully (e.g. increase the overall use of space) and stabilize variance across natural variation in the environment. As in all other experimental and observational studies, the patterns of functional structure reported along the Argentinean coast can have multiple causality [24], [26] and we discuss some of these issues here. Before discussing these specific issues, we need to keep in mind that a basic and general assumption of this, and most published studies, is that local species and functional group abundance represents a time-integrated measure of biological and ecological responses to fluctuating environmental conditions and, thus, a once-off survey captures the ecologically important spatial patterns. Evaluating this assumption requires surveys over multiple years, sufficiently spaced-apart (ideally several years apart) so as to allow for some independence between surveys (e.g. renewal of a large fraction of the dominant individuals). In this manner, the patterns reported here can be visualized as a baseline against which future studies can be compared.

### Coincident Compositional-functional Geographic Discontinuities

Our quantitative analysis of range endpoints for shallow water mollusks and rocky intertidal communities supported the largely descriptive studies that defined three main biogeographical provinces and the position of the transition zones along the Argentinean coast. Unpublished data for decapod crustaceans, though database previously described [40], also support the existence and location of this compositionally-defined transition (M. Fernández, unpublished data).

While patterns of functional group abundance were not generally matched with biogeographical borders, an exception was the abrupt, discontinuous change in the abundance of mid intertidal ephemeral algae, which was strongly and significantly associated with the occurrence of a biogeographical boundary. Indeed, this area is characterized by a sharp discontinuity in macroalgal species richness and taxonomic turnover [31]. The marked higher abundance of ephemerals in the Magellanic Province was largely due to relatively lush cover of thin, fast-growing foliose algae (*Ulva* and *Porphyra*) established atop mussels, which dominated primary space. Therefore, “competitive release” from other sessile species that could exclude them at northern sites is unlikely to account for the observed changes. Benthic grazers appear to have weak or non-significant effects at a nearby site in Cabo Dos Bahias, within the Magellanic Province [41], but no information exists for sites further north, either in the transition zone or Argentinean Province. We did not observe consistent and abrupt increases in abundance (or size, pers. obs.) of grazers north of the Magellanic Province limit, so it is unlikely that herbivory alone is responsible for the abrupt geographic change in ephemeral cover. Experimental manipulations at multiple sites across the different biogeographical provinces are needed to evaluate this proposition. Increased availability of inorganic nutrients is known to stimulate the abundance of ephemeral macroalgae in coastal waters [42], [43]. Given the well-established negative relationship between temperature and nutrient availability in coastal waters [44], [45], [46], the colder sea surface temperatures associated with the subantarctic waters of the Magellanic region may enhance growth, reproduction and/or survival of foliose ephemerals. This explanation suggests that these foliose algae suffer from nutrient limitation under the warmer and assumedly more nutrient-poor conditions to the north, which is in line with trends observed after nutrient enrichment in other parts of the world [43], [47]. The increased abundance of filamentous morphologies (e.g. *Polysiphonia*, *Ceramium*), known to be less sensitive to nutrient poor conditions [47], in the lower shore north of the warmer, saltier waters that mark the thermal front in San Matias Gulf (e.g. north of El Sótano), is consistent, at least in part, with this hypothesis. Unlike most other functional groups, ephemeral algae by their very nature are expected to be susceptible to seasonal variation and thus the documented spatial break in abundance during springtime may be transient, disappearing in late summer and winter months, but should become apparent again over the next spring season. This seasonal variation offers further opportunities to evaluate hypotheses through field experimentation.

Temperature and desiccation stress during aerial conditions is an important factor in controlling species geographical distribution and functional structure in many intertidal organisms [48], [49]. Because of the intensity and persistence of dry winds and high solar radiation, environmental stress has been proposed to be of overriding importance to the structure of intertidal communities along the Argentinean coast [50], [51]. If environmental stress is of overriding importance, we would expect that mid intertidal levels would more clearly show coincident compositional-functional geographical discontinuities than lower levels, where desiccation stress is ameliorated by longer immersion times. Indeed, the coincident change observed occurred in the mid intertidal zone. However, this was observed for only a single functional group (ephemeral algae) and communities across mid shore habitats were more similar among sites than those in the low shore. Thus, physiological stresses during aerial conditions do not appear to play an overriding role, at least not in a simple way, in setting boundaries to both compositional and functional structure of these communities.

### Similarity in Functional Community Structure

The lack of abrupt changes in abundance of invertebrate groups and the relatively weak spatial discrimination observed in mid shore, mussel-dominated communities along the Argentinean coast contrasts sharply with that described for southeastern and northeastern Pacific coasts, where abrupt geographical breaks in mussel abundance are associated with regional discontinuities in onshore larval recruitment rates driven by marked differences in wind-driven upwelling in the northern [15] and southern hemispheres [8], [9]. The comparison with Chile is of particular interest since the filter feeder abundance pattern reflects, in part, the same dominant mussel species, *Perumytilus purpuratus* (Lamarck 1819). This species forms extensive monoculture beds along the central coast of Chile, but at about 32°S and for hundreds of kilometres to the north, mussel beds disappear as an important functional group from the mid zone, despite the fact that several other mussel species coexist with *P. purpuratus* [9]. Along the Argentinean coast, *P. purpuratus* dominates all along the mid shores in the Magellanic Province and has its northern range limit at Pta. Mejillon (site 5). But here, this habitat forming species is replaced by another mussel species, *Brachidontes rodriguezii* (d’Orbigny 1842), with little change in functional abundance in the local communities (see also Adami, M. L., Schwindt, E., Labraga, J. C., Pastorino, G., Tablado, A. and Orensanz, J. M., unpublished manuscript). The geographical persistence in functional group abundance, as observed in mussels, indicates that species with different histories (occupying different geographical areas) converge on traits that maintain similar overall group performance and abundance. The degree to which species are ecologically ‘interchangeable’ over geographical scales has important consequences for predicting functional changes and resilience of local communities to species loss as well as geographical range shifts associated with climate change [52].

We should note that our ability to detect and quantify differences between biogeographical regions may have been hampered by the low number of sites sampled in the Argentinean Province, as well as the fact that both sites were rather far from the transition zone. This sampling limitation was unavoidable due to lack of suitable rocky shore habitat in the region. We found that geographical distance among sites did not correlate with distance in functional group structure at any tidal height, suggesting that the separation of these sites from the rest did not affect our interpretation. The relative scarcity and fragmentation of rocky habitat in the Argentine province may indeed underlie spatial processes (e.g. dispersal and movement) that mediate production and abundance of different functional groups [53], [54] and ultimately impede or enhance between-region differences. In all, these limitations produced by the geography of the continent undoubtedly pose constraints on results at this biogeographical boundary and should be born in mind when evaluating our conclusions.

### Species Richness and “Ecological Performance”

A positive relationship between the number of species that comprise a given functional group (species richness) and total biomass (or other measure of abundance) of the group suggests that, over geographical scales, species richness increases the ability of that group to exploit and deplete resources, such as primary space, food, or nutrients, which has usually been considered an indication of “ecological performance” [24]. A review of over 110 experimental studies has shown that richness is positively associated to standing stock across all functional groups studied and that increased standing stock is generally associated to increased resource depletion [26]. Similar conclusions have been reached regarding other ecosystem functions [20]. Along the Argentinean shores and with the sole exception of filter feeders, we observed generally positive, significant correlations between the biomass (or cover, or density) of individual functional groups and species richness and these relationships were unrelated to biogeographical limits or transition zones. It should be born in mind that increased overall abundance with increasing species richness is not as intuitive or statistically-determined as it might appear. Indeed, high standing stocks and productivity have been commonly associated to species poor systems [55], which led to suggest the existence of a generally negative or unimodal relationship between richness and productivity or overall standing stock [24], [55], [56]. The positive effect of species richness on functional group standing stock can be generated by “complementarity” of species within the functional group, via niche differentiation and facilitation [23], [26], and/or by stochastic community assembly, which causes more speciose communities to have a higher probability of “sampling” [18] a highly productive dominant species from the regional species pool [24], [25]. In experimental manipulations it is unclear to what extent this “sampling effect” of species richness is really an artifact of the experimental design [26]. In our study we cannot tease apart the relative importance of these two mechanisms, but both, complementarity and/or sampling effect, occur under natural variation in these communities.

An inverse relationship between spatial variance and species richness was observed by Loreau et al. [24] and recorded by us at both small (metre) and large (kilometre) scales. Such a relationship is expected if species conforming the functional group are largely uncorrelated over space, which is the basis for the hypothesis of biodiversity as “insurance” [27]. This hypothesis states that even when high diversity is not critical for maintaining ecosystem processes under a constant environment, it can be important for maintaining function under naturally variable conditions. In this manner, biodiversity provides an “insurance” or a buffer against environmental fluctuations because different species have different physiological/ecological responses to these fluctuations, leading to more predictable aggregate community or ecosystem properties [57], [58]. In our geographical evaluation, functional groups with more species exhibit significantly lower biomass variability within sites as well as among sites, suggesting that although species belong to the same functional group, they are sufficiently uncorrelated over space to spatially stabilize standing stock and, potentially, ecosystem function. Little is known about physiological or demographic effects of environmental variation for the species that comprise these functional groups, but resilience is amenable to field determination [59]. Therefore, a detailed and mechanistically oriented investigation of the determinants of richness-variability relations across geographical scales is possible.

Filter feeders were the one functional group that consistently presented no relationship between species richness and biomass or spatial variance in biomass. Filter feeders primarily comprised mussels and, secondarily, barnacles, which probably compete for primary space and collectively often dominate the shore surface. The lack of response in this group may be due to strong overlap of resource use by the different species, causing their ecological performance to be insensitive to species deletions or additions. Previous studies have found that assemblages containing only strong competitors may reduce species responses to environmental variability, weakening or preventing any insurance effect [57], [60]. The fact that the only intertidal barnacle species found in Argentina and included in this functional group is a recent introduction from the northern Hemisphere [61], [62] should also be born in mind when further examining functional structure in this group.

### Conclusions

Our findings allowed us to evaluate general hypotheses about the geographical consistency in the processes that affect species composition and functional structure and provide insight into the importance of species richness within functional groups. The key findings are that the abundance of functional groups can be insensitive to species composition and biogeographical boundaries, and that the richness of species comprising a functional group is positively related with overall biomass and inversely associated with spatial variability in biomass of that assemblage. These findings have important implications for the management and conservation of coastal resources, as the loss of a portion of the species within functional groups would influence not only realized productivity/ecological performance of the group in the community, but also the ability of the group to adjust to environmental fluctuations over local and regional scales.

## Supporting Information

Figure S1 **Species Accumulation plot for the mid intertidal zone at Mar del Plata.**
(TIFF)

Figure S2 **Bray-Curtis ordination plots.** Bray-Curtis ordination plots based on functional group abundances for each of the 20 sites within the two biogeographic regions and transition zone. The first two axes explained 83%, 82%, and 78% of variation on low, mid-low, and mid shores, respectively.
(TIF)

Figure S3 **Total species richness (number of species summed across quadrats) across the low, mid-low, and mid intertidal zones. ND = no data.**
(TIF)

Table S1 **Correlations (Pearson) between a) mean species richness and local abundance (cover or density, see Methods) and b) mean species richness and within-site spatial variation (coefficient of variation, CV, among quadrats) of abundance across the 20 sites surveyed for the low, mid-low, and mid intertidal zones.**
(DOC)
