# Supplementary material for: Biogeographical Boundaries, Functional Group Structure and Diversity of Rocky Shore Communities along the Argentinean Coast
Source: PLoS One. 2012 Nov 16;7(11):e49725. doi: 10.1371/journal.pone.0049725 (PMC3500334; doi:10.1371/journal.pone.0049725)
Supplement: Table S1 — Correlations (Pearson) between a) mean species richness and local abundance (cover or density, see Methods) and b) mean species richness and within-site spatial variation (coefficient of variation, CV, among quadrats) of abundance across the 20 sites surveyed for the low, mid-low, and mid intertidal zones. (DOC) [file pone.0049725.s004.doc]

**Table S1.** Correlations (Pearson) between a) mean species richness and local abundance (cover, density) and b) mean species richness and within-site spatial variation (coefficient of variation, CV, among quadrats) across the 20 sites surveyed for the low, mid-low, and mid intertidal zones.

|  | **Low** | **Mid-Low** | **Mid** |
| --- | --- | --- | --- |
| a) *Species Richness vs. Abundance* | | | |
| Articulated | **0.51*** | **0.61*** | **0.64**** |
| Corticated | **0.81***** | **0.95***** | **NA** |
| Ephemeral | **0.58**** | **0.80***** | **0.76***** |
| Filter feeder | 0.30 | 0.39 | 0.25 |
| Grazer | **0.81***** | **0.58*** | 0.43 |
| Predator | **0.89***** | **0.78***** | **0.92***** |
| Scavenger | **0.51*** | **NA** | **NA** |
| b) *Species Richness vs. CV Abundance* | | | |
| Articulated | **-0.58*** | **-0.71**** | **-0,81***** |
| Corticated | -0.46 | **-0.83**** | **NA** |
| Ephemeral | **-0.64**** | **-0.62*** | **-0.72***** |
| Filter feeder | -0.69 | -0.42 | 0.06 |
| Grazer | **-0,81***** | **-0.82***** | **-0.79***** |
| Predator | **-0.78**** | **-0.84**** | **-0.93***** |
| Scavenger | **-0.66*** | **NA** | **NA** |

Bold values indicate significance at α = 0.05.

* p < 0.05, ** p < 0.01, *** p < 0.001

NA indicates where the functional group was too scarce in a given tidal level for meaningful correlations.
